# Chronic phantom pain as a rare phenomenon after gender-affirming surgery: a systematic comparison study

**DOI:** 10.1097/PR9.0000000000001328

**Published:** 2025-09-18

**Authors:** Barbara Schlisio, Andreas Kugel, Christian Grasshoff

**Affiliations:** Department of Anesthesiology and Intensive Care Medicine, Eberhard-Karls- University, Tuebingen, Germany

**Keywords:** Gender-affirming surgery, Phantom pain, Chronic postsurgical pain

## Abstract

The occurrence of phantom pain after surgical removal of male reproductive organs is a potential outcome. Indications for surgery may predict the probability of phantom pain after the procedure. These may vary between individuals undergoing tumor orchiectomy and those undergoing gender-affirming surgery. This study investigates the incidence of phantom pain after gender-affirming surgery. A retrospective questionnaire-based study was conducted with 55 transgender women who had undergone gender-affirming surgery, and the results were compared with those of 54 men who had undergone tumor orchiectomy, serving as a control group. The frequency, severity, risk factors, and characteristics of phantom pain in the testes and penis, painless phantom sensations in the genital area, and chronic postsurgical pain incidence were assessed. Phantom pain and painless phantom sensations in the testes were more frequent after tumor orchiectomy than after gender-affirming surgery. Transgender women did not experience persistent phantom pain after gender-affirming surgery. Chronic postsurgical pain incidence did not differ between the 2 groups. Preexisting genital pain affected the risk of chronic postsurgical pain in the tumor orchiectomy group but not the risk of phantom pain. In this study, we assessed whether transgender women experienced phantom pain after undergoing gender-affirming surgery. It appears that transgender women are less prone to phantom pain after gender-affirming surgery than are men after inguinal tumor orchiectomy. This may be attributed to alterations in the central representation of the unaccepted sex organs in the somatosensory cortex.

## Abstract 2

The relationship between transgender identity and neuroplasticity suggests a potential influence on the occurrence of phantom limb pain after gender-affirming surgery.

## 1. Introduction

Phantom pain after limb amputation is a common and well-studied phenomenon. It occurs in approximately 60% to 80% of amputees.^17^ Loss of visceral organs is also associated with phantom pain. Phantom pain and sensation have both been described as common phenomena after mastectomy and rectal amputation.^1,5,15^ Puhse et al. demonstrated an incidence of 53% for phantom sensation and 25% for phantom pain in the testicular region one year after inguinal tumor orchiectomy.^19^

Male-to-female gender-affirming surgery is performed on transgender women under conditions entirely different from those involved in the oncological removal of the testicle. Unlike tumor removal surgery, this procedure is explicitly desired by patients. Studies on outcomes after gender-affirming surgery (GAC) have mostly focused on aspects of quality of life, such as psychological well-being, sexuality, and life satisfaction.^16^ Phantom pain has not been systematically investigated. A recent review by Bishop et al. on dysfunction after GAC included studies on pain during sexual intercourse, muscle pain in the shoulder girdle after mastectomy, and nonspecific pelvic pain.^4^ Phantom and chronic postoperative pain have received little attention. To our knowledge, this study is the first attempt to address this gap in the literature. We aimed to investigate the incidence of phantom and chronic postoperative pain after male-to-female gender-affirming surgery and compare their outcomes with those of patients who had undergone inguinal tumor orchiectomy.

## 2. Methods

### 2.1. Study design and patients

The trial was approved by the Ethics Committee of the University of Tübingen in August 2014 (project number 611/2013BO2) and registered at ClinicalTrials.gov under the identifier NCT04538170. A comparative study was conducted between 2 patient groups: those who had undergone GAC and those who had undergone inguinal orchiectomy for a testicular tumor (ORC). Patients were recruited from Tübingen University Urological Hospital and Munich Bogenhausen Urological Clinic. The gender-affirming surgery consisted of removing the testicles, the spermatic cord, and the corpora cavernosa, as well as vaginoplasty.

**Figure 1.**
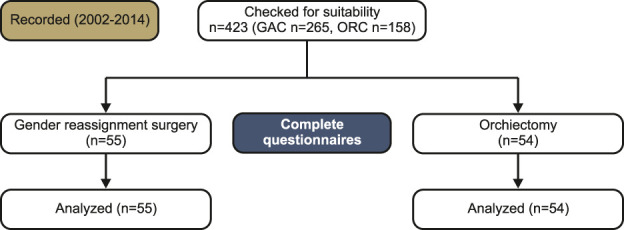
Chart of study population.

A total of 265 transgender women who had undergone surgery between 2002 and 2014 and 158 men who had undergone tumor orchiectomy between 2010 and 2014 were contacted. Completed questionnaires from 55 participants in the GAC group and 54 participants in the ORC group were evaluated, with response rates of 20.75% and 34.18%, respectively. A diagram of the study population is shown in Figure 1.

The inclusion criteria for the study were an interval of at least 6 months between surgery and completion of the questionnaire, as well as German language proficiency.

### 2.2. Questionnaire

Patients received a questionnaire that assessed the following categories: demographics, current phantom pain and phantom pain over time, preoperative and postoperative pain status, psychological pain processing parameters, sexual life, and urologic outcomes. The assessment of pain is conducted using the German Pain Questionnaire, a standardised and validated instrument. The questionnaire serves as a fundamental data source and a crucial element in the quality assurance of chronic pain patients in Germany.^18^ Phantom sensations without pain and pain management with perioperative epidural anesthesia were also assessed. The pain intensity was assessed at different time points using an 11-point numeric rating scale (NRS).

The Pain Detect Questionnaire for the detection of neuropathic pain was integrated into the questionnaire on pain quality.^10^

### 2.3. Data analysis

Preliminary work by Puhse et al. showed that approximately 25% of patients reported phantom pain after inguinal tumor orchiectomy.^19^ Based on these results, a statistically significant sample size was determined. A difference of 20% between the GAC and ORC groups was considered relevant. Power was set at 80% and the significance level at 5%, requiring a minimum of 40 transgender women to be recruited, a condition that was fulfilled. Group comparisons were performed for qualitative data using the χ^2^ test. The means of quantitative data were compared using a *t* test for independent samples. The significance level was set at *P* = 0.05.

## 3. Results

### 3.1. Patient demographics

At the time of surgery, the mean age did not significantly differ between both patient groups (ORC: 40.7 ± 11.4 years, range: 18–66 years; GAC: 44.3 ± 9.9 years, range: 25–64 years; *P* = 0.081). The time between data collection and surgery averaged 3.0 years in the ORC group and 4.5 years in the GAC group, which was significantly longer (*P* = 0.001). Table 1 summarizes the demographic data of both patient groups.

**Table 1:** Patient demographic.

	ORC		GAC		*P*
	N	%	N	%	
Marital status					
Single	12	22.2%	17	30.9%	0.387
Divorced	3	5.6%	13	23.6%	**0.013**
Married	32	59.3%	14	25.5%	**<0.001**
Widower	0	0.0%	1	1.8%	1.000
Permanent partner	7	13.0%	10	18.2%	0.599
Professional status					
In training	5	9.6%	2	3.6%	0.262
Working	43	82.7%	36	65.5%	**0.050**
Retired	3	5.8%	7	12.7%	0.322
Early retiree	1	1.9%	2	3.6%	1.000
Unemployed	0	0.0%	7	12.7%	**0.013**
Homemaker	0	0.0%	1	1.8%	1.000
Education					
No degree	0	0.0%	1	1.9%	1.000
Elementary school	10	18.5%	16	29.6%	0.260
Middle school	4	7.4%	11	20.4%	0.093
High school	33	61.1%	19	35.2%	**0.012**
Other	7	13.0%	7	13.0%	1.000
Confession					
None	13	24.1%	27	49.1%	**0.010**
Roman Catholic	13	24.1%	10	18.2%	0.489
Protestant	23	42.6%	16	29.1%	0.165
Nondenominational	1	1.9%	0	0.0%	0.495
Muslim	2	3.7%	0	0.0%	0.243
Other	2	3.7%	2	3.6%	1.000

ORC, inguinal tumor orchiectomy; GAC, gender-affirming surgery. The significant *P*-values are indicated by entries in bold.

### 3.2. Preoperative pain status

Patients were assessed for preexisting pain, pain location, and associated pain-related impairments before planned GAC. A total of 22 patients in the ORC group (40.7%) and 3 patients in the GAC group (5.6%) reported preexisting preoperative pain (*P* < 0.001). The pain was primarily localized in the testicular and inguinal regions. A comprehensive list of preoperative pain statuses is shown in Table 2.

**Table 2:** Preoperative pain status.

	ORC		GAC		*P*
	N	%	N	%	
Preoperative pain in the genitals	22	40.74	3	5.56	**<0.001**
Pain right testicle	11	20.37	1	1.81	
Pain left testicle	8	14.81	1	1.81	
Pain groin area	5	9.26	0	0.00	
Pain glans	0	0.00	1	1.81	
Unknown pain location	2	3.70	0	0.00	
Impaired sexual function due to pain	4	7.40	0	0.00	
Medical care due to pain	6	11.11	1	1.81	

Absolute and relative numbers in relation to the total patient cohort. Multiple answers allowed for pain localization.

ORC, orchidectomy; GAC gender-affirming surgery.

Four men in the ORC group and none in the GAC group reported sexual function impairments. Pain treatment was administered to 6 men in the ORC group and one transgender woman in the GAC group. The average preoperative pain intensity was 3.36 (±2.77, n = 22) in the ORC group compared with 1.67 (±1.53, n = 3) in the GAC group (*P* = 0.315). Maximum pain intensity amounted to 5.14 (±2.71, n = 22) in the ORC group and 7.00 (±1.00, n = 3) in the GAC group (*P* = 0.315). The self-assessment of general sensitivity to pain on a scale of 0 (insensitive) to 10 (very sensitive) yielded an almost identical mean of 4.48 (ORC, n = 54) and 4.44 (GAC, n = 55) in both groups (*P* = 0.867).

### 3.3. Phantom pain in testicles and penis

**Figure 2.**
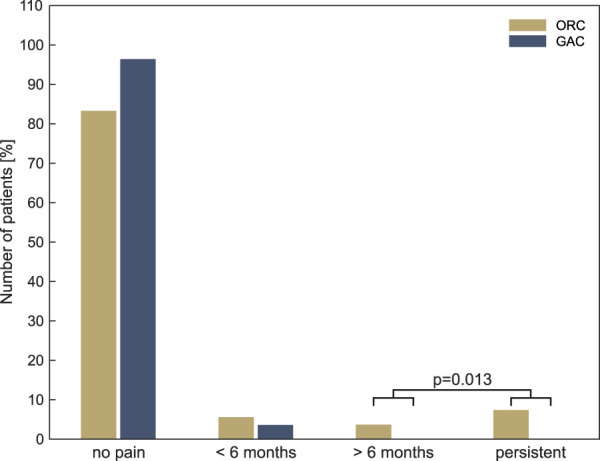
Phantom pain in the testicular area in GAC and ORC patients over time. Chronic phantom pain was defined as pain that persisted for more than 6 months. A comparative analysis was conducted between the ORC group, which included individuals with pain persisting for more than 6 months and individuals with persistent phantom pain at the time of the survey, and the transgender women group (*P* = 0.013). GAC, gender-affirming surgery; ORC, inguinal tumor orchiectomy.

Chronic phantom pain was defined as pain that persisted for more than 6 months. A comparative analysis was conducted between the ORC group, which included individuals with phantom pain persisting for more than 6 months and persistent phantom pain at the time of the survey, and the transgender women group. According to this definition, six men in the ORC group (11.1%) reported chronic phantom pain in the testicles, while no transgender women in the GAC group reported chronic phantom pain in the testicles. This difference was statistically significant (*P* = 0.013). Figure 2 shows the incidence and persistence of phantom pain in the testicular area over time. Four transgender women reported phantom pain in the former penis. In 3 cases (5.5%), this was experienced for up to 6 months postoperatively, while in one case (1.8%), it persisted for more than 6 months. None of the transgender women reported persistent phantom pain in the penile region. When describing the character of phantom pain over time, “pain attacks and pain-free in between” dominated in both groups (ORC: 7 of 8 men with phantom pain, 87.5%; GAC: 2 of 3 transgender women, 66.7%), followed by “continuous pain with pain attacks” (ORC: one man, 12.5%, GAC: one transgender woman, 33.3%). “Continuous pain with slight fluctuations” and “pain attacks, pain in between” were not reported.

A correlation has been observed between the age of the patient and the incidence of phantom pain, although the relationship is found to be very weak. The Pearson correlation coefficient r is −0.093.

### 3.4. Persistent phantom sensations of the testicles and penis

**Figure 3.**
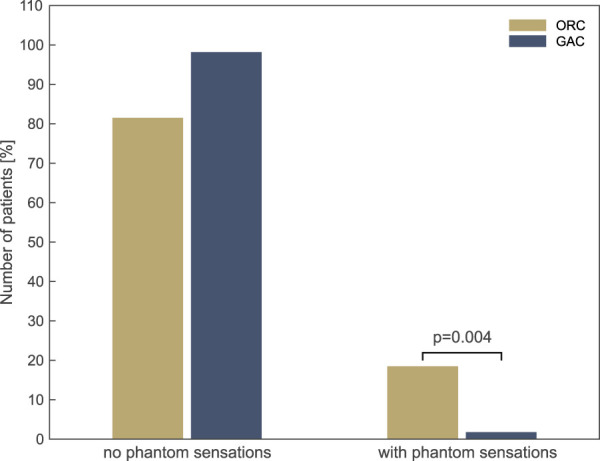
Painless phantom sensation of the testicles and penis.

Ten men (18.5%) in the ORC group reported a nonpainful postoperative phantom sensation in the testicular region, while only one transgender woman (1.8%) in the GAC group reported the same. The occurrence of phantom sensation in the testicle was significantly more prevalent (*P* = 0.004) after inguinal tumor orchiectomy than after GAC (Fig. 3).

### 3.5. Persistent postoperative pain

Persistent postoperative pain was defined as genital pain that persisted for more than 6 months. A total of 13 patients in the ORC group (24.1%) and 15 patients in the GAC group (27.3%) reported chronic pain. There was no significant difference in the frequency of chronic postoperative pain between the 2 groups (*P* = 0.827). The average pain intensity of patients with persistent postoperative pain was 4.31 ± 2.98 in the ORC group and 3.00 ± 2.99 in the GAC group on the NRS scale, where 10 reflects the maximum possible pain. The maximum pain intensity of patients with persistent postoperative pain was 5.31 ± 3.17 in the ORC group and 5.50 ± 2.32 in the GAC group. The main locations of pain after GAC surgery were reported to be in the neovaginal area (n = 10, 18.2%) and the clitoral area (n = 8, 14.4%), followed by pain in the labia (n = 5, 9.1%) and pain in the groin (n = 3, 5.5%). One patient in this group could not precisely localize the pain. After inguinal tumor orchiectomy, patients most frequently reported groin pain.

### 3.6. Influence of preoperative pain status on chronic phantom pain and chronic postoperative pain

Preoperative pain did not increase the incidence of phantom pain after GAC (*P* = 1.00). However, this was not the case for chronic postoperative pain. Of the 22 men in the ORC group with preoperative pain, 10 (45.5%) experienced chronic postoperative pain, compared with only 3 (9.4%) of the 32 men without preoperative pain. This difference was statistically significant (*P* = 0.004). None of the 3 transgender women with preoperative pain in the GAC group had chronic postoperative pain, while 15 (28.8%) of the 52 transgender women without preoperative pain developed persistent local pain in the surgical area beyond 6 months.

### 3.7. Sexual function disorder

Sexual function disorders due to phantom sensations were reported by 2 men in the ORC group and one transgender woman in the GAC group.

## 4. Discussion

Phantom pain in the testicles was more frequently reported in orchiectomized men than in transgender women after GAC. Furthermore, phantom sensations were significantly more prevalent in orchiectomized men. These findings support the hypothesis that phantom pain is significantly less common after GAC than after inguinal tumor orchiectomy, despite the fact that GAC is a surgical procedure involving a considerable degree of trauma. To explain this phenomenon, it is necessary to conclude that the observed differences are due to either morphological or functional reasons.

In transgender women, there is a discrepancy between their assigned male gender and individually perceived female identity, which characteristically evokes a desire for biological transition. Male sex organs are often perceived as incongruous with gender identity or as foreign bodies. This led to the hypothesis that the cortical sensory representations of male sex organs may have diminished or been functionally eliminated. The question of whether it is possible to perform neuronal mapping of a “typical” male or female brain is also a pivotal issue in transgender research. Evidence shows that there seem to be morphological differences between male and female populations with respect to gray and white matter weight and structures.^22^ Rametti et al. sought to verify the white matter differences between transgender individuals and control female and male individuals using diffusion-weighted MRI imaging.^21^ White matter microstructural patterns differed in nearly all fascicles compared with control females or males, and resembled the desired sex. Similarly, the structures of the nucleus striae terminalis and the third interstitial nuclear area of the anterior hypothalamus differed, which significantly influenced sexual behavior.^11,13^ Smith et al. posited that transgender women display neuromorphological similarities to the desired female sex, yet they are not wholly identical to the female sex. However, based on the neuroanatomical characteristics of the brains of transgender men and women, it is not possible to definitively determine their biological sex.^24^ This means that, in addition to neuroanatomical characteristics, secondary functional change and adaptation of neuronal imprinting in the direction of the desired gender must also be considered.

An illustrative example is the alteration of these functional abilities due to hormone therapy. In a study conducted by Burke et al., it was demonstrated that the administration of male hormone therapy to transgender males resulted in enhanced spatial imagination. Conversely, transgender women who received estrogen therapy exhibited augmented verbal communicative abilities.^7^ This process, which can also be paraphrased as plasticity, presumably affects the mapping of sex organs in the somatosensory cortex. Functional reorganization that occurs in the sensorimotor cortex after tissue amputation may serve as a measurable factor in the development of phantom limb pain, as evidenced by numerous research papers.^3^ Reorganization is defined as the displacement of neighboring representations into the primary somatosensory cortex (S1) of the deafferented cortical area until it is reoccupied. The degree of measurable reorganization has been shown to directly correlate with the intensity of phantom pain.^2,9^

Because the transgender women in our study had no phantom pain in the testicular area, the representation area of the rejected sexual organs must be either absent or very minimal. This means that reorganization processes rarely occur. Several questions remain unanswered: whether transgender women have a somatosensory representation of their male genitalia, what factors contribute to the reduction in the representational area of the rejected genitalia in transgender women, and when this development occurs. Regarding the sensorimotor cortex and its influence on the development of phantom sensation, Ramachandran and McGeoch postulated an innate, fixed framework for the internal body image, which they termed the “hardware”.^20^ According to their hypothesis, the neural investment in organs is primarily located in the somatosensory cortex. To illustrate this, they described the observation of phantom sensations in individuals born without a corresponding limb.^23^ A comparable observation was documented by Brugger et al.,^6^ based on a 44-year-old female patient who was born without legs and forearms. The results of functional magnetic resonance imaging and transcranial magnetic stimulation of the sensorimotor cortex demonstrated that body parts that were not physically present could be represented in the cortex. However, in this example, it can be postulated that the extremities are not rejected as foreign. This neurological “hardware”^20^ might be modified in patients with gender incongruence.

Applying this to our study, this means that transgender women may have the body of a male with appropriate somatosensory representation, but this is functionally or epigenetically suppressed or diminished—making phantom pain in the penis or testes very unlikely after gender-affirming surgery. Conversely, these transgender women could experience a female phantom breast, or a transgender man could experience a phantom penis, even before the respective surgical gender-affirming, if the cortical representation is functionally or epigenetically variable. This phenomenon was demonstrated in an observational study by Langer et al. Almost 50% of the transgender people or gender-diverse people surveyed described transphantoms (ie, a phantom penis experienced by a transgender man or a phantom vagina experienced by a transgender woman).^14^

Our sample showed that transgender women did not experience chronic phantom pain. Only one transgender woman described a mere phantom sensation in the testicles after GAC. In the control group, the phantom pain incidence of 11.1% was lower than that in the study by Puhse et al.^19^ In this study, 25% of patients in the study population of 238 patients reported phantom pain after inguinal tumor orchiectomy, and 15.8% described phantom sensation. In comparison, the incidence of phantom sensation in our study was 18.5% in the tumor orchiectomy group.

The occurrence of phantom pain in the penis of transgender women was uncommon and did not extent beyond the observation period. The finding can be explained by reference to the specific surgical procedure involved in the operation. In the procedure known as orchiectomy, the nerves supplying the testicle are severed, resulting in the occurrence of free nerve endings and the potential for the development of neuroma. Conversely, the dorsal nerve of the penis, which is surgically embedded in the neovagina, remains intact. This is likely to reduce the risk of phantom pain in the penis, because the tissue innervation is maintained.

In addition, persistent general postoperative pain represents a significant outcome parameter that has the capacity to impair the quality of life on a long-term, permanent basis. In our study, there was no significant difference in the incidence of chronic postoperative pain between female patients and those who underwent tumor-related orchiectomy (*P* = 0.827). Haroutiunian et al. found a high prevalence of neuropathic pain in their meta-analysis on chronic postoperative pain.^12^ In addition, there was a suspicion that neuropathic pain might play a substantial role, given that, despite the use of a nerve-sparing dissection approach, injury to minor peripheral nerve endings is challenging to avoid. Interestingly, with very low overall pain detection scores, we diagnosed neuropathic pain in only a few patients. In terms of the estimated subjective overall pain sensitivity, measured on a scale of 0 to 10, the results were similar in both groups. Questioning this aspect of individual pain sensitivity was important to highlight any differences between the groups and narrow it down to a random bias for chronic and phantom limb pain. Fillingim et al. reported different perceived pain intensities from 0 to 100 NRS to a heat stimulus of 48° in 321 healthy subjects.^8^ In this study, the mean pain intensity was measured at 71.8 with a variation range of 4 to 100 NRS. Moreover, occurrence of phantom pain could be linked to other risk factors, including preexisting pain disorders. To reduce this confounding factor, preexisting chronic pain conditions were recorded. This potential confounding factor was found to have no statistically significant influence on the study's results.

Limitations: The study was conducted as a pilot study. A longitudinal study design would have provided more precise information regarding the temporal dynamics of pain. Furthermore, the utilization of face-to-face interaction could have mitigated potential misunderstandings. For subsequent studies, the incorporation of functional MRI should be considered. Furthermore, the implementation of a QST analysis would have been advantageous in the characterization of the pain. A further limitation is the response rate of the questionnaires and the selection bias. It was observed that a number of the transgender women were no longer contactable at their registered addresses, presumably because they wished to change their living environment, a course of action that is understandable for this population. The low response rate is another potential area for improvement, and the use of email reminders might be beneficial in this regard. It is unfortunate that this form of follow-up contact was not desired. A notable limitation is the selection bias, which is presumably based on voluntary participation in the study and the study design. Only transgender women with uncomplicated surgery could have responded. Conversely, it could be hypothesized that transgender women experiencing phantom pain or chronic pain postoperatively would be inclined to share their experiences and, consequently, the adverse outcomes of the surgery for scientific evaluation. However, it is possible that the inclusion of a disproportionately high number of male subjects afflicted with phantom pain subsequent to inguinal tumor orchiectomy in the study population may have resulted in an overestimation of phantom pain incidence.

In retrospect, it must be noted that observational studies are subject to selection bias, which is a significant disadvantage. Nevertheless, they are useful for generating hypotheses.

The perception of pain can be modified. Transsexuality demonstrated these modifications and their potential impact on phantom pain. Neuroplasticity, which refers to the capacity of the human brain to adapt and change, may have implications for the development of novel therapeutic approaches. Futuristic but conceivable treatments could include virtual reality glasses to diminish or eliminate the sensory perception of painful body regions.

## Disclosures

The authors have no conflict of interest to declare.
